# Alteration of Connective Tissue Growth Factor (CTGF) Expression in Orbital Fibroblasts from Patients with Graves’ Ophthalmopathy

**DOI:** 10.1371/journal.pone.0143514

**Published:** 2015-11-24

**Authors:** Chieh-Chih Tsai, Shi-Bei Wu, Pei-Chen Chang, Yau-Huei Wei

**Affiliations:** 1 Department of Ophthalmology, Taipei Veterans General Hospital, Taipei 112, Taiwan; 2 Department of Medicine, National Yang-Ming University, Taipei 112, Taiwan; 3 Department of Biochemistry and Molecular Biology, National Yang-Ming University, Taipei 112, Taiwan; 4 Institute of Clinical Medicine, National Yang-Ming University, Taipei 112, Taiwan; 5 Department of Medicine, Mackay Medical College, New Taipei City 252, Taiwan; 6 Institute of Biomedical Sciences, Mackay Medical College, New Taipei City 252, Taiwan; IPATIMUP/Faculty of Medicine of the University of Porto, PORTUGAL

## Abstract

Graves’ ophthalmopathy (GO) is a disfiguring and sometimes blinding disease, which is characterized by inflammation and swelling of orbital tissues, with fibrosis and adipogenesis being predominant features. The aim of this study is to investigate whether the expression levels of fibrosis-related genes, especially that of connective tissue growth factor (CTGF), are altered in orbital fibroblasts of patients with GO. The role of oxidative stress in the regulation of CTGF expression in GO orbital fibroblasts is also examined. By a SYBR Green-based real time quantitative PCR (RT-QPCR), we demonstrated that the mRNA expression levels of fibronectin, apolipoprotein J, and CTGF in cultured orbital fibroblasts from patients with GO were significantly higher than those of age-matched normal controls (*p* = 0.007, 0.037, and 0.002, respectively). In addition, the protein expression levels of fibronectin, apolipoprotein J, and CTGF analyzed by Western blot were also significantly higher in GO orbital fibroblasts (*p* = 0.046, 0.032, and 0.008, respectively) as compared with the control. Furthermore, after treatment of orbital fibroblasts with a sub-lethal dose of hydrogen peroxide (200 μM H_2_O_2_), we found that the H_2_O_2_-induced increase of CTGF expression was more pronounced in the GO orbital fibroblasts as compared with those in normal controls (20% vs. 7%, *p* = 0.007). Importantly, pre-incubation with antioxidants including *N*-acetylcysteine (NAC) and vitamin C, respectively, resulted in significant attenuation of the induction of CTGF in GO orbital fibroblasts in response to H_2_O_2_ (*p* = 0.004 and 0.015, respectively). Taken together, we suggest that oxidative stress plays a role in the alteration of the expression of CTGF in GO orbital fibroblasts that may contribute to the pathogenesis and progression of GO. Antioxidants may be used in combination with the therapeutic agents for effective treatment of GO.

## Introduction

Graves’ ophthalmopathy (GO), also known as thyroid-associated ophthalmopathy or thyroid eye disease, is clinically evident in 10–45% of patients with Grave’s disease [1]. GO may compromise the life quality of affected individuals and even cause vision impairment in severe cases. The clinical course of GO is characterized by both inflammation and tissue remodeling [2]. Inflammation often dominates the early event of GO, followed by profound remodeling of orbital connective tissue, including aberrant accumulation of extracellular matrix macromolecules and fibrosis [3]. Although fibrosis represents a relative quiescent stage in the natural course of GO, it may cause much of the substantial morbidity of the patients including persistent lid retraction, restrictive strabismus, proptosis, exposure keratopathy, and optic nerve compression [4]. In addition, patients with inactive sclerotic disease are often unresponsive to conventional immunosuppressive treatment and require surgical intervention. Current understanding of the inflammatory process of GO has been rapidly expanding, however, only a few studies have addressed the sclerotic stage of this disease [5–7]. Elucidation of the molecular mechanisms that initiate and regulate the process of fibrosis in GO is crucial for the development of novel treatments. Over the past few years, several fibrogenic factors including fibronectin, apolipoprotein J, and connective tissue growth factor (CTGF) have been identified. Among them, CTGF has been shown to be most critical. It is a cysteine-rich protein (~40 kD) secreted by various cell types and is often co-expressed with transforming growth factor-β (TGF-β) [8]. They exert biological functions by binding to specific receptors and induce cell migration, proliferation, differentiation, extracellular matrix synthesis, and tissue fibrosis [9]. CTGF has been shown to be substantially involved in the pathogenesis of various fibrotic disorders such as liver, heart and kidney fibrosis [10–12]. Recently, CTGF has also been shown to play a role in ocular fibrosis process of human lens epithelial cells, corneal fibroblasts, tenon's capsule fibroblasts and trabecular meshwork cells, and in proliferative vitreoretinopathy membrane [13–17]. However, the role of CTGF in the fibrosis process of GO has not been clarified.

CTGF expression is regulated by various extracellular molecules such as TGF-β and dexamethasone [18, 19]. Besides, reactive oxygen species (ROS) have also been found to be an important inducer of CTGF expression in both *in vivo* and *in vitro* studies [20–22]. Perturbation of the intracellular levels of oxidants or antioxidants can lead to the buildup of ROS, which may cause various degenerative diseases, including tissue fibrosis [23, 24]. In addition, different lines of evidence has been accumulated to substantiate that oxidative stress is involved in the pathogenesis of GO [25–28]. Our previous studies have demonstrated that compared with normal fibroblasts GO orbital fibroblasts are much more sensitive to oxidative stress elicited by hydrogen peroxide (H_2_O_2_) and are important targets in the development of GO [29, 30]. Based on these findings, we investigated whether the protein expression levels of fibrosis-related genes, including that of CTGF, are up-regulated in the primary culture of orbital fibroblasts. We demonstrated that exogenous oxidative stress elicited by H_2_O_2_ treatment could induce the expression of CTGF protein in the orbital fibroblasts. We also examined whether or not oxidative stress-induced synthesis of CTGF in the GO orbital fibroblasts could be reduced by pretreatment of the cells with antioxidants.

## Materials and Methods

### Tissues acquisition and cell culture

All specimens were collected in accordance to the Declaration of Helsinki and with informed consent of the patients. These protocols were approved by the Institutional Review Board of Taipei Veterans General Hospital (IRB number: 2014-04-004AC). Written informed consent was also obtained from each of the donors for the clinical specimens used in this study. The primary cultures of orbital fibroblasts were established from surgical specimens of 5 patients with GO (G1-G5) during decompression surgery (one men and four women; mean age: 36.5 years) and from apparently normal orbital tissues in five age- and sex-matched patients (N1-N5) who received surgery for noninflammatory conditions (one man and four women; mean age: 35.8 years). All GO patients received methimazole and achieved stable euthyroidism for at least 6 months before surgery and were in the inactive stage of GO. Exclusion criteria include ocular diseases other than GO, alcohol drinking, regular ingestion of drugs or antioxidants, and pregnancy. Individuals suffering from chronic or acute diseases, such as diabetes mellitus, hyperlipidemia, diseases of the lung, liver, or kidney, cancer, other endocrine dysfunction, and immunological or inflammatory disorders were also excluded. In addition, all study subjects had not received specific treatment (systemic steroids or radiotherapy) for GO. Briefly, the orbital tissues were minced aseptically in phosphate-buffered saline (PBS, pH 7.3), and then incubated with a sterile solution containing 0.5% collagenase and dispase (Sigma-Aldrich Chemical Co., St. Louis, MO, USA) for 24 hr at 37°C in an incubator filled with an atmosphere of 5% CO_2_ [29, 30]. The digested orbital tissues were pelleted by centrifugation at 1,000 g, and then resuspended in Dulbecco’s Modified Eagle’s Medium (DMEM, purchased from Gibco Life Technologies, Gaithersburg, MD, USA) containing 10% fetal bovine serum (FBS) and a cocktail of antibiotics (Biological Industries, Kibbutz Beit Haemek, Israel), which was composed of 100 U/ml penicillin G and 100 μg/ml streptomycin sulfate (Biological Industries, Kibbutz Beit Haemek, Israel). Cultured orbital fibroblasts were used between the 3rd and 5th passages and the cell cultures at the same passage number were used for the same set of experiments.

### Real-time reverse transcriptase-polymerase chain reaction

The expression levels of fibrosis-related genes were determined by SYBR Green-based real time quantitative PCR (RT-QPCR). Briefly, the total cellular RNA from orbital fibroblasts lysates was extracted with a chloroform solution after adding the TRIZol reagent (Sigma-Aldrich Chemical Co, MO, USA), and was then precipitated with isopropanol solution followed by dissolution of RNA in DEPC-H_2_O. An aliquot of 5 μg RNA was reverse-transcribed to cDNA with the Ready-to-Go RT-PCR kit (Amersham Biosciences, Uppsala, Sweden) at 42°C for at least 16 hr. Quantitative RT-PCR was performed using the SYBR Green Master kit (Sigma-Aldrich) according to the manufacturer’s instructions [31]. The primer pairs are 5’-ATTGGCAATGAGCGGTTC-3’ and 5’-GGATGCCACAGGACTCCAT-3’ for β-actin, 5’-CTGCAGGCTAGAGAAGCAGAG-3’ and 5’-GATGCACTTTTTGCCCTTCT-3’ for CTGF, 5’-CTGGCCGAAAATACATTGTAA-3’ and 5’-CCACAGTCGGGTCAGGAG-3’ for fibronectin, and 5’-GGACATCCACTTCCACAGC-3’ and 5’-GGTCATCGTCGCCTTCTC-3’ for apolipoprotein J, respectively. The mRNA expression level of each gene in the orbital fibroblasts was normalized with the mRNA level of the β-actin gene, respectively.

### Western blot analysis

Approximately 1x10^7^orbital fibroblasts were pelleted, washed with PBS, and then re-suspended in the lysis buffer containing 50 mM Hepes (pH 7.4), 4 mM EDTA, 2 mM EGTA, 1 mM Na_3_VO_2_, 1 mM NaF, 1% Triton X-100, and an aliquot of complete protease inhibitors (Roche Inc., Mannheim, Germany). The suspension was incubated on ice for 20 min and then centrifuged at 10,000g for another 20 min at 4°C. The supernatant was collected and the protein concentration was determined by the method of Bradford (Bio-Rad Laboratories, Hercules, CA, USA). An aliquot of 50 μg proteins was separated on 10% SDS-PAGE and blotted onto a piece of the PVDF membrane (Amersham-Pharmacia Biotech Inc., Buckinghamshire, UK). After blocking by 5% skim milk in the TBST buffer (50 mM Tris-HCl, 150 mM NaCl, 0.1% Tween 20, pH 7.4) at room temperature for 1 hr, the membrane was incubated for another 1 hr with the primary antibody at room temperature. After washing three times with the TBST, the blot was incubated with a horseradish peroxidase (HRP)-conjugated secondary antibody for 1 hr at room temperature. An enhanced chemiluminescence detection kit (Amersham-Pharmacia Biotech Inc., Buckinghamshire, UK) was used to detect the protein signals with a Fuji X-ray film (Fuji Film Corp., Tokyo, Japan), and the intensities of signals were quantified by ImageScanner III with LabScan 6.0 software (GE Healthcare BioSciences Corp., Piscataway, NJ, USA). The antibodies against CTGF (SC-14939) and β-actin (#A1978) were purchased from Santa Cruse Biotechnology Inc. (CA, USA) and Sigma-Aldrich Chemical Co. (MO, USA), respectively. The expression level of CTGF in the orbital fibroblasts was normalized by that of β-actin and is presented as a relative value compared to that of N1. All data are expressed as mean ± SD of the results obtained from three independent experiments.

### Measurement of CTGF secretion by ELISA

By using the ELISA kit (#SK00726) purchased from Adipo Bioscience, Inc. (Santa Clara, CA, USA), we determined the CTGF protein levels in orbital fibroblasts from GO patients and normal subjects, respectively [16]. About 5×10^5^ orbital fibroblasts were subjected to analysis for the release of CTGF in each set of experiments. Briefly, the cultured medium was collected and centrifuged at 12,000 g at 4°C and the aliquots were immediately assayed according to the manufacture’s suggestion. The standards of CTGF were used in the range of 62.5–1000 pg/ml and the results were normalized by the cell numbers and expressed as pg/10^6^ cells. All experiments were performed in triplicate with three different sets of orbital fibroblast cultures.

### Treatment of orbital fibroblasts with H_2_O_2_ and antioxidants

After treatment with 200 μM H_2_O_2_ for 1 hr, the cultured orbital fibroblasts were washed with PBS buffer (pH 7.3) twice and were re-incubated with the complete medium for another 24 hr. On the other hand, we pre-treated orbital fibroblasts with 1 mM N-acetylcysteine (NAC) and 2 mM vitamin C for 1 hr followed by treatment of the cells with of H_2_O_2_.

### Statistical analysis

Statistical analysis was performed by using the Microsoft Excel 2010 statistical package and SigmaPlot software version 12.3 (Systat Software Inc., San Jose, CA, USA). The data are presented as means ± standard deviation (SD) of the results obtained from three independent experiments. The significance level of the difference between the control and the experimental groups was determined by the Student's t test. A difference was considered statistically significant when the *p value<0.05 and **p value<0.01, respectively.

## Results

### Up-regulation of mRNA and protein expression of fibrosis-related genes in GO orbital fibroblasts

**Fig 1:**
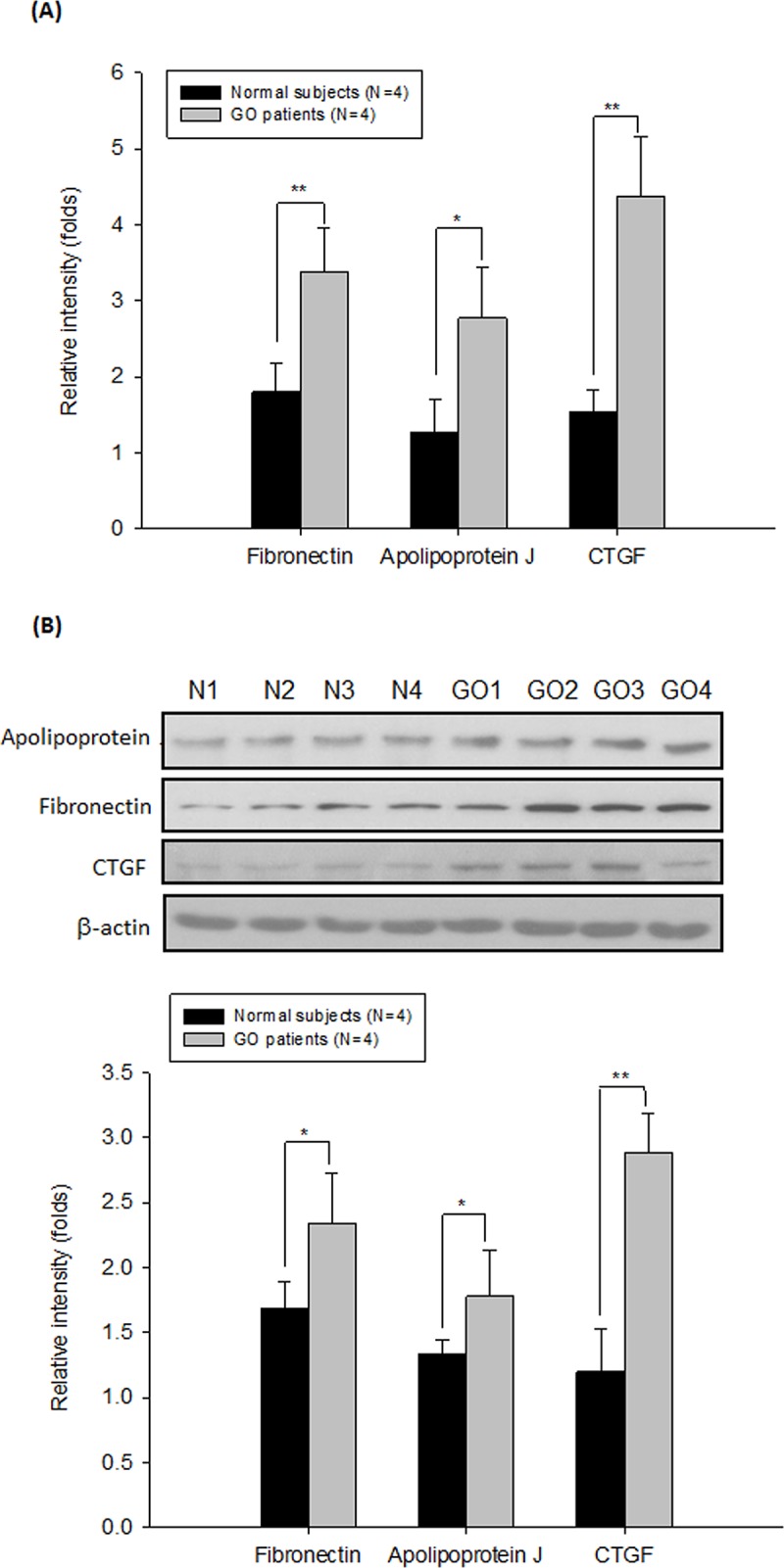
Increase in the expression of fibrosis-related genes in the primary cultures of GO orbital fibroblasts. (A) The mRNA and protein expression levels of fibrosis-related genes including apolipoprotein J, fibronectin and CTGF were determined by RT-PCR and (B) by Western blot analysis in the primary cultures of orbital fibroblasts from GO patients (GO1-GO4) and age-matched controls (N1-N4), respectively. By densitometric analysis of three independent Western blots, the protein expression levels of apolipoprotein J, fibronectin and CTGF were normalized to the corresponding β-actin expression level. The representative histogram was constructed on the basis of the mean values of proteins expression levels in the primary cultures of orbital fibroblasts. Data are presented as means ± SD of the results from three independent experiments (*, p< 0.05, ** p< 0.01 vs. the indicated group).

**Fig 2:**
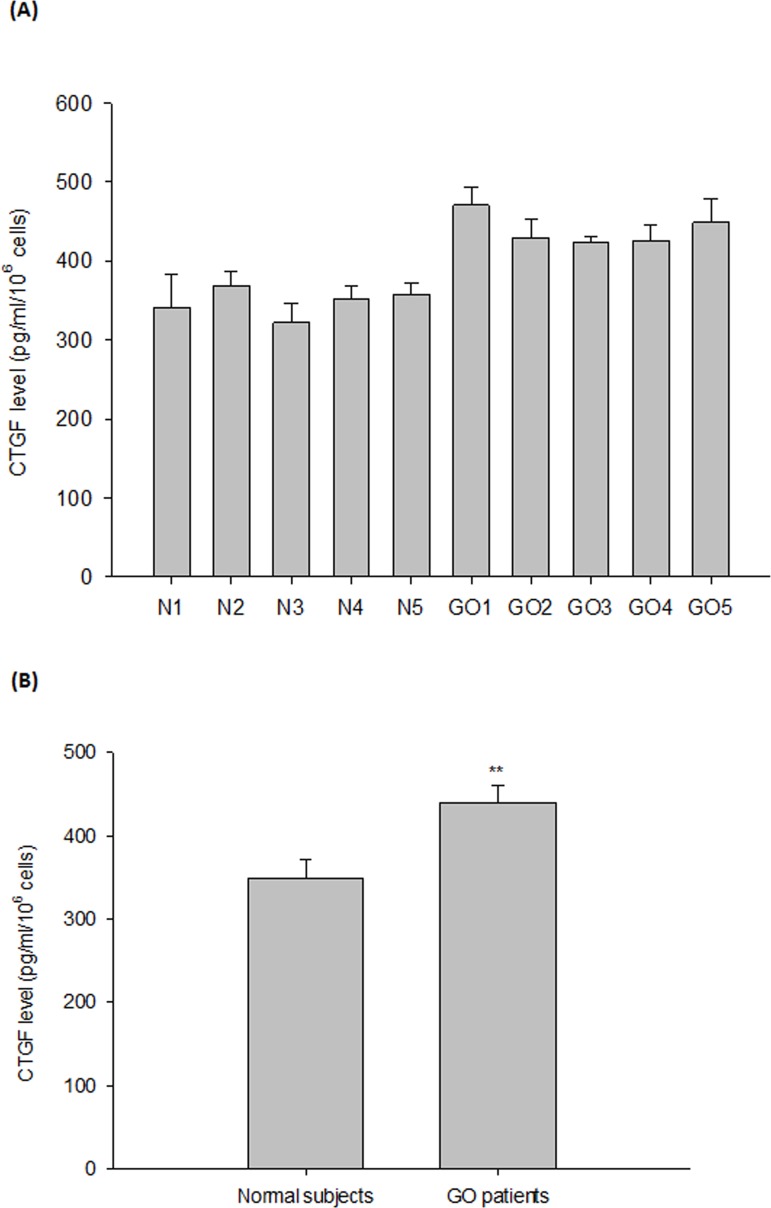
Increase in the release of CTGF in the primary cultures of GO orbital fibroblasts as compared to those of controls. (A) By a CTGF ELISA kit, the basal levels of the release of CTGF in the culture medium of orbital fibroblasts were determined. (B) The mean value of the release of CTGF in the cultured medium was determined from 5 GO orbital fibroblasts and 5 age-matched controls. Data are presented as means ± SD of the results from three independent experiments (** p< 0.01 vs. the indicated group).

By a SYBR-based RT-PCR, we first observed that the protein expression levels of fibronectin, apolipoprotein J and CTGF were increased in the GO orbital fibroblasts as compared to those of normal subjects (p = 0.007, 0.037, and 0.002, respectively) (Fig 1A). In addition, the protein expression levels of fibronectin, apolipoprotein J and CTGF evaluated by Western blot were also significantly higher in the GO orbital fibroblasts as compared to those of normal subjects, and the difference was more pronounced for CTGF expression (p = 0.046, 0.032, and 0.008, respectively) (Fig 1B). On the other hand, by using the ELISA analysis, we further noted that the secretion of CTGF protein in the cultured medium from the GO orbital fibroblasts was substantially higher than that of normal subjects (p = 0.003) (Fig 2).

### ROS-induced changes of CTGF secretion in orbital fibroblasts

**Fig 3:**
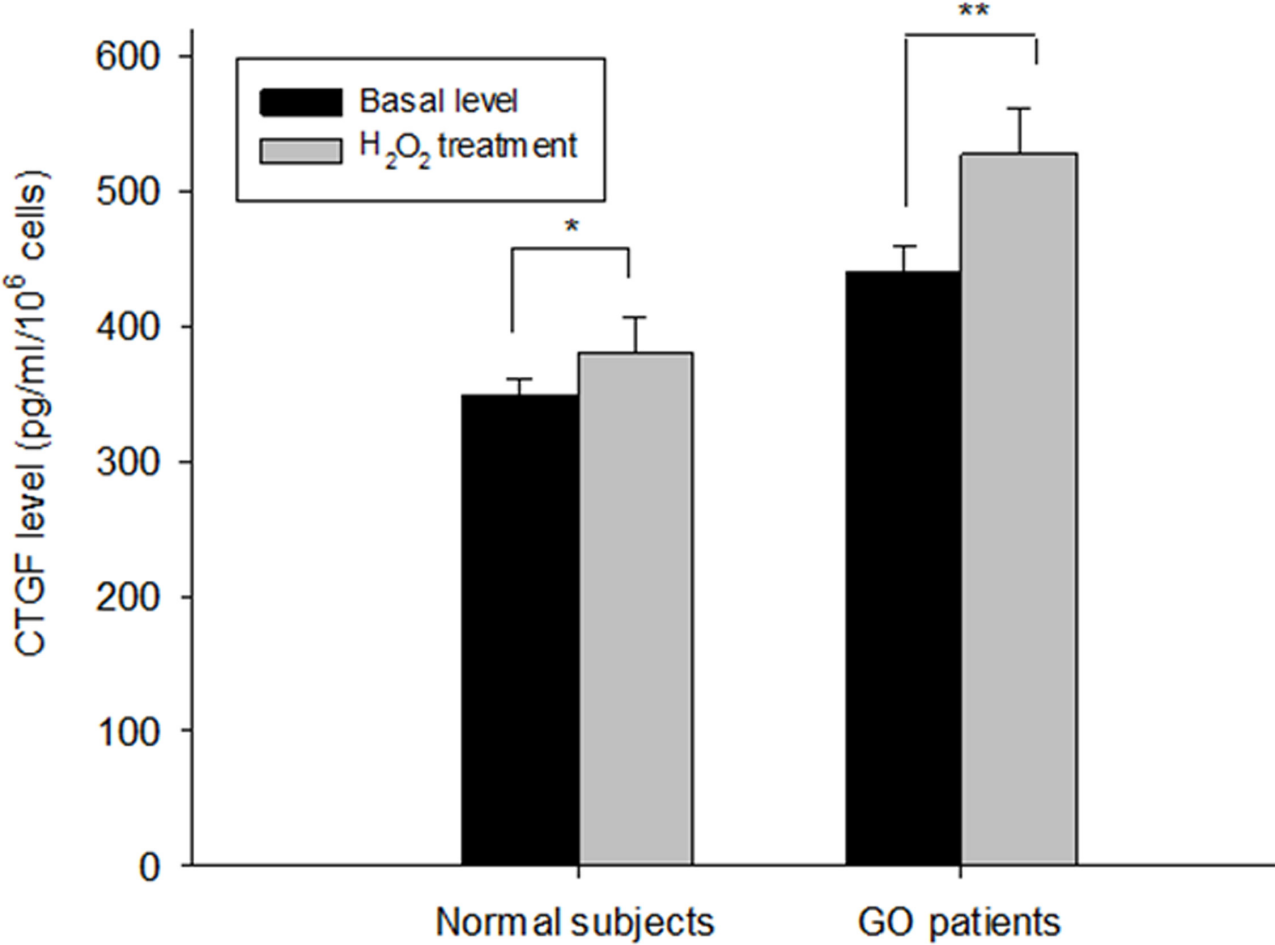
Susceptibility to H_2_O_2_-induced release of CTGF in the primary culture of GO orbital fibroblasts. After treatment of orbital fibroblasts with 200 μM H_2_O_2_ for 1 hr, the cells were washed with the PBS buffer, followed by incubation in a complete medium for another 24 hr. By a CTGF ELISA kit, the H_2_O_2_-induced release of CTGF in the cultured medium was assayed in the primary cultures of orbital fibroblasts from 5 GO patients and those of age-matched controls, respectively. The representative histogram was constructed as a bar-graph on the basis of the results from three independent experiments. Data are presented as means ± SD of the results from three independent experiments (*, p< 0.05, ** p< 0.01 vs. the indicated group).

After induction of oxidative stress by treatment of orbital fibroblasts with 200 μM H_2_O_2_ for 1 hr, the secretion of CTGF was elevated both in the normal (p = 0.015) and GO (p = 0.003) orbital fibroblasts (Fig 3). However, the increase of CTGF in response to H_2_O_2_ treatment was more pronounced in the GO orbital fibroblasts compared with those in normal controls (20% vs. 7%, p = 0.007).

### Inhibition of ROS-induced CTGF expression by antioxidants

**Fig 4:**
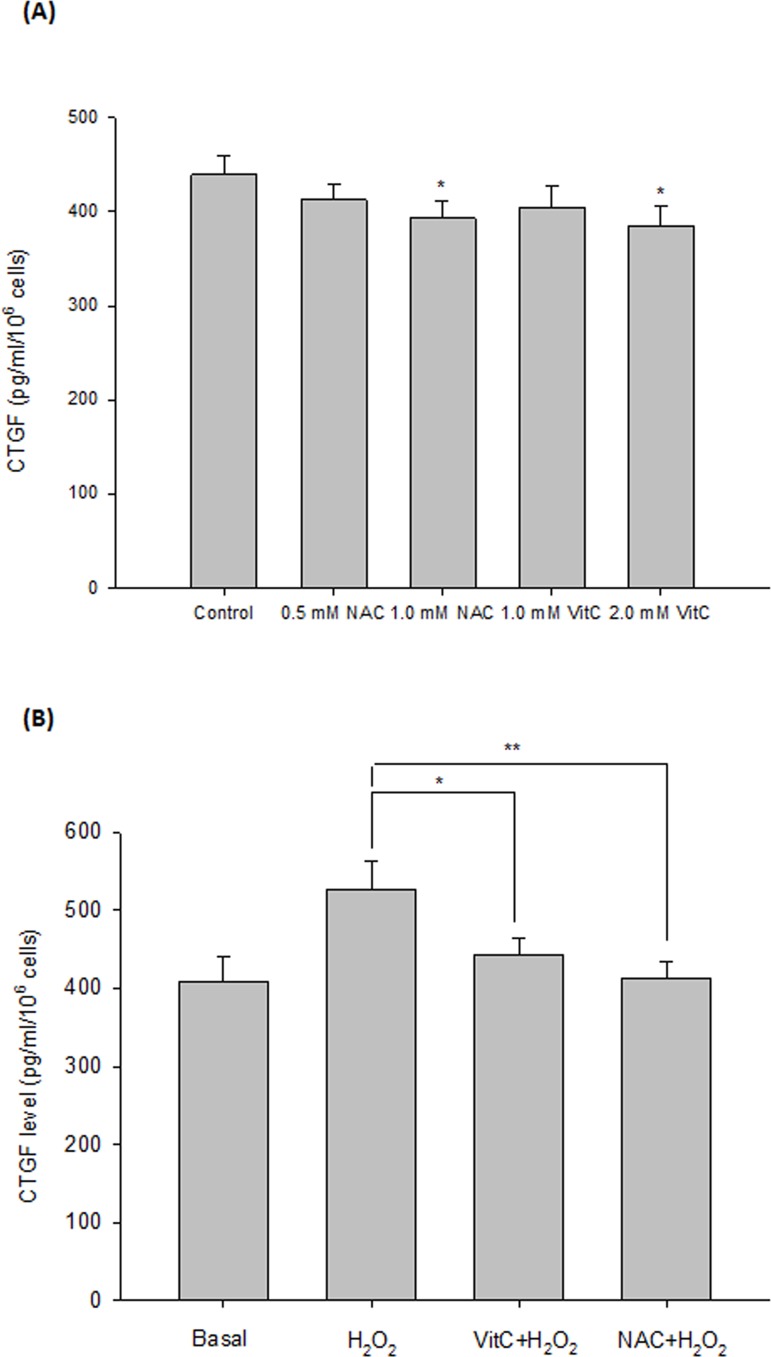
Inhibition of H_2_O_2_-induced CTGF expression by pre-treatment of GO orbital fibroblasts with antioxidants. By using a CTGF ELISA kit, the expression of CTGF was determined in the primary cultures of orbital fibroblasts from 5 GO patients. (A) The GO orbital fibroblasts were incubated with antioxidants (without H_2_O_2_) including NAC (0.5 mM and 1.0 mM) and VitC (1 mM and 2 mM) for 24 hr, respectively. (B) Orbital fibroblasts were pre-incubated with antioxidants including 1 mM NAC and 2 mM vitamin C (Vit C), respectively, followed by treatment with 200 μM H_2_O_2_ for 1 hr. The cells were then washed with the PBS buffer and were cultured with a complete medium for another 24 hr. The mean value was constructed as a bar-graph on the basis of the results from three independent experiments, and data are presented as means ± SD of the results from three independent experiments (*p< 0.05, **p< 0.01 vs. the indicated group).

To investigate whether the effect of oxidative stress could be blocked by antioxidants, we pre-treated orbital fibroblasts with NAC and vitamin C, respectively, for 1 hr followed by the H_2_O_2_ treatment. The results showed that pre-incubation of cells with 1 mM NAC or 2 mM vitamin C could significantly inhibit the 200 μM H_2_O_2_-induced elevation of CTGF production in the GO orbital fibroblasts (p = 0.004 and 0.015, respectively) (Fig 4).

## Discussion

Orbital fibroblasts, the major target cells in the pathogenesis of GO, play an important role in not only the early inflammatory process but also the subsequent tissue remodeling of GO [32]. This is the first study to provide evidence of the increase of proteins expression levels of fibrosis-related genes, especially CTGF, in the primary cultures of GO orbital fibroblasts as compared with those of normal controls. More importantly, we found that the production of CTGF by GO orbital fibroblasts was increased after exposure to exogenous oxidative stress. Pre-treatment with antioxidants such as NAC and vitamin C could confer significant protection against the influence of exogenous H_2_O_2_ on CTGF production.

Overproduction of CTGF has been observed in a variety of fibrotic disorders affecting multiple organ systems, including several ocular fibrosis diseases [13–17]. CTGF is an extracellular matrix-modulating protein, which is thought to be the downstream mediator of TGF-β, and can enhance many biological effects of TGF-β [33, 34]. Notably, CTGF is critical for TGF-β-mediated fibroblast-myofibroblast transdifferentiation and subsequent deposition of extracellular matrix [35, 36]. In addition, CTGF has been proved to have independent fibrogenic functions. Zhang et al. [15] showed that CTGF can induce phenotypic transition into myofibroblast on human tenon’s fibroblasts individually and significantly promoted their proliferation and synthesis of extracellular matrix molecules such as collagen type I and fibronectin. It was demonstrated that the myofibroblast differentiation in Thy-1^+^ fibroblasts is associated with tissue remodeling and fibrosis of GO [37]. A recent study further revealed that antisense inhibition to CTGF *in vivo* could abrogate wound fibrosis by reduction of the myofibroblasts differentiation and the transcription of several extracellular matrix proteins including tissue inhibitor of metalloproteinase-1 (TIMP-1) [38]. The disruption in the balance between matrix metalloproteinases and TIMPs could contribute to a pro-fibrotic microenvironment in GO [7, 39]. These findings suggest that elevated expression of CTGF in GO orbital fibroblasts might alter the natural course of tissue remodeling in GO. However, the underlying molecular mechanism warrants further investigation. In the clinical setting, TGF-β has been considered as one of the therapeutic targets to inhibit fibrosis. Substantially, we have disclosed in a previous study that TGF-β is elevated in response to oxidative stress challenge in GO orbital fibroblasts [30]. However, TGF-β is an upstream and initial factor that exerts a broad spectrum of physiological functions and thus efforts to modulate its expression have been limited by concerns about treatment specificity and adverse effects [40]. It has been suggested that the downstream signaling molecules of TGF-β such as CTGF can provide a much safer and more effective target in the treatment of these fibrotic disorders [15, 38].

There is abundant evidence showing that oxidative stress is involved in the pathogenesis of GO [25–27, 41]. We have previously reported that oxidative stress elicited more pronounced response of ROS metabolism and proliferation in GO orbital fibroblasts [29, 30]. The present study provided further evidence to substantiate that oxidative stress can induce CTGF expression in orbital fibroblasts, especially in those from GO patients. These findings are consistent with those of Park and Matsuda [21, 22], who showed that oxidative stress caused upregulation of CTGF expression in human lens epithelial cells and retinal pigment epithelial cells, respectively. Oxidative stress is also one of the primary factors that can induce pathological fibrosis [23, 24]. GO orbital fibroblasts have been shown to exhibit discrete phenotypes and had exaggerated responses to proinflammatory cytokines [42]. Taken together, our previous and present studies clearly suggest that GO orbital fibroblasts displayed exaggerated responses to oxidative stress, which might be associated with cellular proliferation, oxidative damage, and tissue remodeling in GO patients [27, 29, 30]. These peculiar characteristics of GO orbital fibroblasts may underscore their susceptibility to oxidative damage in this eye disease. Most importantly, we demonstrated in this study that the induction of CTGF by oxidative stress can be attenuated by antioxidants in GO orbital fibroblasts. Recently, it was reported that selenium could be successfully applied in GO patients, and the therapeutic effect of selenium was attributed, at least partly, to its antioxidant effects [28]. Together, previous reports and our findings in this study have substantiated the potential of antioxidants in the treatment of GO.

The present study provided evidence, for the first time, to substantiate that the protein expression levels of fibrosis-related genes, especial CTGF, are elevated in GO orbital fibroblasts. In addition, the expression of CTGF in orbital fibroblasts can be modulated by oxidative stress. The findings obtained in this study provided some clues for the development of potential therapeutic agents for better control of tissue remodeling in GO.
